# CDKN2A and MTAP Are Useful Biomarkers Detectable by Droplet Digital PCR in Malignant Pleural Mesothelioma: A Potential Alternative Method in Diagnosis Compared to Fluorescence *In Situ* Hybridisation

**DOI:** 10.3389/fonc.2020.579327

**Published:** 2020-11-13

**Authors:** Yuen Yee Cheng, Man Lee Yuen, Emma M. Rath, Ben Johnson, Ling Zhuang, Ta-kun Yu, Vesna Aleksova, Anthony Linton, Steven Kao, Candice Julie Clarke, Brian C. McCaughan, Ken Takahashi, Kenneth Lee

**Affiliations:** ^1^ Asbestos Diseases Research Institute, Concord, NSW, Australia; ^2^ Giannoulatou Laboratory, Victor Chang Cardiac Research Institute, Darlinghurst, NSW, Australia; ^3^ Faculty of Medicine, University of New South Wales, Sydney, NSW, Australia; ^4^ Concord Repatriation General Hospital, School of Medicine, University of Sydney, Sydney, NSW, Australia; ^5^ Chris O’Brien Life House, School of Medicine, University of Sydney, Sydney, NSW, Australia; ^6^ Anatomical Pathology Department, NSW Health Pathology, Concord Repatriation General Hospital, Sydney, NSW, Australia; ^7^ Sydney Cardiothoracic Surgeons, RPA Medical Centre, Sydney, NSW, Australia

**Keywords:** methylthioadenosine phosphorylase, CDKN2A, fluorescence *in situ* hybridization, droplet digital PCR, malignant pleural mesothelioma

## Abstract

**Background:**

The diagnosis of malignant pleural mesothelioma (MPM) can be difficult, in part due to the difficulty in distinguishing between MPM and reactive mesothelial hyperplasia (RMH). The tumor suppressor gene, CDKN2A, is frequently silenced by epigenetic mechanisms in many cancers; in the case of MPM it is mostly silenced *via* genomic deletion. Co-deletion of the CDKN2A and methylthioadenosine phosphorylase (MTAP) genes has been researched extensively and discovered to be a highly specific characteristic of MPM. Most studies have used FISH to detect the deletion of CDKN2A and IHC for MTAP as a surrogate for this. In this study, we aim to investigate and validate droplet digital PCR (ddPCR) as an emerging alternative and efficient testing method in diagnosing MPM, by particularly emphasizing on the loss of MTAP and CDKN2A.

**Methods:**

This study included 75 formalin fixed paraffin embedded (FFPE) MPM tissue, and 12 normal pleural tissue and 10 RMH as control. Additionally, primary MPM cell lines and normal pleural samples were used as biomarker detection controls, as established in our previous publication. All FFPE specimens were processed to isolate the DNA, that was subsequently used for ddPCR detection of CDKN2A and MTAP. FFPE samples were also analyzed by fluorescence *in situ* hybridization (FISH) for CDKN2A and MTAP deletion, and for MTAP IHC expression. Concordance of IHC and ddPCR with FISH were studied in these samples.

**Results:**

95% and 82% of cases showed co-deletion of both MTAP and CDKN2A when determined by FISH and ddPCR respectively. ddPCR has a sensitivity of 72% and specificity of 100% in detecting CDKN2A homozygous loss in MPM. ddPCR also has a concordance rate of 92% with FISH in detecting homozygous loss of CDKN2A. MTAP IHC was 68% sensitive and 100% specific for detecting CDKN2A homozygous loss in MPM when these losses were determined by ddPCR.

**Conclusion:**

Our study confirms that MTAP is often co-deleted with CDKN2A in MPM. Our in-house designed ddPCR assays for MTAP and CDKN2A are useful in differentiating MPM from RMH, and is highly concordant with FISH that is currently used in diagnosing MPM. ddPCR detection of these genetic losses can potentially be utilized as an alternative method in the diagnosis of MPM and for the future development of a less-invasive MPM-specific detection technique on MPM tumor tissue DNA.

## Background

Malignant pleural mesothelioma (MPM) is an aggressive malignancy caused by exposure to asbestos. The prognosis is poor with limited effective treatment options (1, 2). MPM is strongly linked to previous asbestos exposure (3) and asbestiform minerals such as erionite and fluoro-edenite (4). Most MPM patients are diagnosed at a late stage due to non-specific symptoms presenting at early stages coupled with the long latency period between asbestos exposure and the development of MPM. The current gold standard for a definitive MPM diagnosis requires the collection of adequate tissue specimen, such as image guided core biopsy, video-assisted thoracoscopy guided biopsy or cytology. Delays or errors in diagnosis potentially hinder treatment intervention which can adversely affect the patients’ survival and quality-of-life (QoL). Therefore accurate diagnosis is essential for prognostication and management (5).

Currently, more than 15 biomarkers are used in the clinical setting to diagnose MPM, and newer platforms are constantly being developed in order to improve the efficiency, sensitivity and specificity of the testing method. Ideally, the new testing platform would also require material obtained from less invasive procedures unlike the current gold standard tissue biopsy, which is required for histomorphologic assessment, concurrent use of certain biomarkers on the obtained tissue by immunohistochemistry (IHC) and/or fluorescence *in situ* hybridization (FISH) in order to reach a definitive diagnosis. The diagnostic algorithm of MPM begins with a morphologic assessment of a cytology or histology specimen, and the use of several biomarkers such as pan *cytokeratin*, *Calretinin*, *CK5/6*, *Thrombomodulin*(CD141), *HBME-1, WT-1, D2-40, EMA, CEA, B72.3 (TAG-72), BG8, CD15, TTF-1, MOC31, claudin-4*, and *BerEP4 (HEA)* by immunohistochemistry (IHC) on formalin fixed (6), paraffin embedded (FFPE) or cell block to differentiate mesothelial from epithelial lineage. The aforementioned biomarkers are by no means an exhaustive list. If the tumor is confirmed to be mesothelial in lineage, but lacks demonstrable evidence of invasion into adjacent soft tissue or viscera, then subsequent biomarkers are used to further assist in differentiating MPM from benign reactive mesothelial hyperplasia (RMH). Recently BAP1 and MTAP IHC have been used for this purpose with reasonably good sensitivity and specificity. However for equivocal cases, FISH for homozygous loss of CDKN2A (7–9) is often used to further differentiate between the two. We have previously shown that BAP1 and CDKN2A loss are frequent in MPM (7). Hwang *et al.*, also indicated that the loss of CDKN2A by FISH and BAP1 by IHC are specific biomarkers for MPM diagnosis (10, 11). In addition, the MTAP gene, located on chromosome 9p21 which codes for the MTAP protein methylthioadenosine phosphorylase, has also been reported to be co-deleted with CDKN2A in MPM (12). As a result of this finding, recent studies have demonstrated that MTAP IHC loss is a good surrogate for CDKN2A homozygous loss (13). This has implications for clinical practice as IHC is often a more convenient, rapid and economical test to perform than FISH (13). Therefore, the use of MTAP IHC has proven to be an additional useful option in practice. Further studies highlighted superior sensitivity and specificity when FISH for CDKN2A deletion is used in combination with MTAP and BAP1 IHC (8, 14). However, there are certain limitations in FISH testing, including the fact that it is expensive, has a relatively longer turnaround time, and requires highly trained staff to perform and interpret.

The droplet digital PCR (ddPCR) technique, based on water-oil emulsion droplet technology, is an emerging PCR method for nucleic acid detection that has a strong potential to become the next-generation biomarker detection platform (15). ddPCR provided higher sensitivity and precision in molecular diagnostics for pathogens such as *hepatitis B virus* (15), *human immunodeficiency virus* (HIV) (16), *chlamydia trachomatis* (17) and *chromosomally integrated human herpes virus 6* (18). We have previously reported ddPCR to be a useful method in analyzing and detecting the loss of CDKN2A in MPM (7). This present study aims to utilize the Asbestos Disease Research Institute’s (ADRI) extensive MPM collection to re-validate the ability of ddPCR to detect the loss of CDKN2A, and the prevalence of MTAP co-deletion in MPM. This study also aims to assess the concordance between ddPCR and FISH in detecting CDKN2A homozygous loss. Finally, we aim to assess the correlation of MTAP IHC with the genetic changes of MTAP (genetic retention and homozygous loss) and with CDKN2A homozygous loss when determined by ddPCR in MPM.

## Methods and Materials

### Immunohistochemical Analysis of MPM Tissue Sections and Established Cell Line Blocks for MTAP

All patients gave informed written consent and the project was approved by the Human Research Ethics Committees at Concord Repatriation General Hospital. All samples obtained from previously diagnosed MPM patients were reviewed by an experienced pathologist (KL) with MPM-specific IHC markers before the experiments were carried out. Immunohistochemical analysis of MTAP (Abnova, H00004507-M01, 2G4 clone; 1/300 dilution) in MPM tissue samples was performed as described previously (7). Briefly, MPM cells and MPM tissue were processed for formalin fixed paraffin embedded (FFPE) block and were sectioned at 4 *μ*m thickness for IHC analysis. Diagnostic laboratory procedures related to the diagnosis of these cases were performed in an accredited human medical testing laboratory (ISO 15189). The method of scoring for each antibody in each case was conducted in accordance with the usual clinical diagnostic practice and was performed by an experienced pathologist. A positive result was defined by a complete loss (absence of IHC cytoplasmic staining in tumor cells) or partial loss (reduced IHC cytoplasmic staining or heterogeneous staining) as compared to retained staining of internal control lymphocytes. A negative result was defined by a retained staining (no loss in the IHC cytoplasmic staining of tumor cells) as compared with the retained staining of the internal control lymphocytes. The expression of MTAP IHC was distinctly binary where loss of cytoplasmic expression is complete, and retained expression was cytoplasmic retention by the whole tumor population. MTAP IHC result was noted as defined above.

### FISH Analysis of MTAP and CDKN2A Genes

Fluorescence *in situ* hybridization (FISH) of MTAP and CDKN2A was performed as described previously (7), but with the additional inclusion of an MTAP-specific probe. Briefly, a *CEP9* Spectrum Green–labeled probe, a Spectrum Orange–labeled, *CDKN2A* locus-specific probe (Cat. 05J51-001, Abbott Molecular), and an aqua-dUTP-labeled MTAP specific probe (MTAP-20-AQ Empire Genomics) were used. Nuclei were counterstained with DAPI/antifade (Vysis). Tissue and cell blocks were hybridized and stained following the procedure described in our previous publication (7). Appropriate control tissue for FISH assay included normal lung pleural tissue sections and reactive pleural fluid cell block (n=10) as a negative control, and sections of mesothelioma previously identified as carrying CDKN2A deletion as a positive control. Analyses were performed by an experienced pathologist with a fluorescence microscope (Axio M2, ZEISS) equipped with filter sets with single- and dual-band exciters for Aqua, Spectrum Green, Spectrum Orange, and DAPI (UV 360 nm). The histologic areas previously selected on the hematoxylin-eosin–stained sections were identified on the FISH-treated slides. Several areas within the tumor is selected for FISH analysis to eliminate selection bias as much as possible. Only individual and well-delineated cells were scored. Overlapping cells were excluded from the analysis. At least 100 cells were scored for each mesothelioma case. Cells with a homozygous deletion were defined by the absence of both red (CDKN2A) signal and aqua signal for the MTAP in the presence of at least one green chromosome 9 signal (*CEP9*). Cells with a hemizygous deletion were defined by the presence of one 9p21 signal, and one or two *CEP9* signals. The cut off values were established by methods previously described (7). The cut off value was the mean percentage + 3 SD using normal mesothelial cell nuclei. We established a cut off value of above 15% of tumor cells with a homozygous deletion as being a homozygous deleted tumor, and greater than 40% of tumor cells with hemizygous deletion as a hemizygous deleted tumor. For comparing FISH counts with ddPCR, each FISH homozygous cell contributes 2 counts and each FISH hemizygous cell contributes 1 count.

### DNA Isolation From FFPE and Cell Line Samples

Genomic DNA was extracted from MPM FFPE samples using the DNA minikit (Qiagen, Valencia, CA, USA) following manufacturer’s instructions with minor modification. Briefly, 2x20 µm FFPE scrolls were collected in each tube and dewaxed with xylene twice following two times 100% ethanol wash. Dried tissue was added to ATL buffer and subsequent steps were carried out according to the manufacturer’s instructions.

### CDKN2A and MTAP Genomic Loss Were Analyzed for Copy Number Variation Using ddPCR

Primers for the amplification of the genomic region of MTAP, *CDKN2A* and E2 were designed (**Supplementary Table 1**) and optimized using ddPCR Probe (Bio-Rad) according to manufacturer’s recommendations. Genomic DNA from MPM FFPE tissues and MPM cell lines were used as a template for ddPCR analysis. ddPCR reaction mixtures were assembled using 2x Probe ddPCR Supermix (Bio-Rad) and primer and probe concentration of 200 nM in a total reaction volume 20 μl. Reactions were dispensed into each well of the droplet generator DG8 cartridge (Bio-Rad). Each oil compartment of the cartridge was filled with 70 μl of droplet generation oil for Probe (Bio-Rad), and approximately 15,000 to 20,000 droplets were generated at each well with use of the droplet generator (Bio-Rad QX200). The entire droplet emulsion volume (40 μl) was transferred to a 96-well PCR plate (Bio-Rad). The plate was then heat sealed with a pierceable foil in the PX1 PCR Plate Sealer (Bio-Rad), and placed in the thermocycler (Bio-Rad T1000). The optimal thermal cycling conditions were used: 95°C for 5 min; 35 cycles of 95°C for 30 s, 60°C for 30 s, 72°C for 1 min; and a final step at 72°C for 1 min. The cycled droplets were read individually with the QX200 droplet-reader (Bio-Rad), and analyzed with QuantaSoft droplet reader software, version 1.7 (Bio-Rad). The error reported for a single well was the Poisson 95% confidence interval. No template controls (NTC) were used to monitor contaminations and primer-dimer formation, and to aid the determination of the cut-off threshold. Copy number for each genomic region was calculated upon normalization to the included reference gene E2 (two copies retained per cell; ie. ddPCR copy number = (ddPCR copy number for gene of interest/ddPCR copy number for E2 control gene) x 2; where the multiplication by 2 is to account for each E2 count representing two alleles per cell). Homozygous deletion was considered in cases where little or no detection of the target genomic region was determined and a distinctive E2 population was apparent. The criteria for assigning homozygous, hemizygous or retained to the sample based on ddPCR counts was previously established with another batch of samples. The following criteria accounts for the tumor percentage (tumor cells vs all cells), as determined by H&E. For samples with greater than 85% tumor percentage, a ddPCR copy number of 0 to 0.4 = homozygous, 0.4 to 1.5 = hemizygous, and 1.5 to 2 = retained. For 60% to 85% tumor percentage, a ddPCR copy number of 0 to 0.7 = homozygous, 0.7 to 1.6 = hemizygous, and 1.6 to 2 = retained. For 35% to 60% tumor percentage, a ddPCR copy number of 0 to 0.8 = homozygous, 0.8 to 1.8 = hemizygous, and 1.8 to 2 = retained. For tumor percentage below 35%, ddPCR of 0 to 1.2 = homozygous, 1.2 to 1.8 = hemizygous, and 1.8 to 2 = retained. The control samples utilized for our ddPCR assays for MTAP and CDKN2A are MeT5A (human immortalized non-cancer mesothelial cell line) and MSTO (human mesothelioma cell line). Previous work has established that MeT5A has retention of both MTAP and CDKN2A (7), and MSTO has homozygous deletion of CDKN2A (7) and hemizygous deletion of MTAP. The ddPCR results correctly identified a retention of MTAP and CDKN2A for MeT5A, and deletion of these genes for MSTO. In addition, ddPCR for CDKN2A and MTAP was performed on 12 normal pleural tissue samples.

### Statistics

Statistical analyses were carried out using IBM SPSS software version 25 and R (19). Spearman’s rank correlation was used to calculate correlations between datasets that were not visually normally distributed. Spearman’s rank correlation was used to calculate correlations between FISH counts for CDKN2A and MTAP, and between ddPCR copy numbers for CDKN2A and MTAP. Spearman’s rank correlation was used to calculate correlations between FISH counts and ddPCR copy number for a given gene, and in this case a negative correlation is interpreted as a concordant correlation as when there is gene loss, the FISH counts are high (number of tumor cells enumerated that showed the loss of either or both genes) and the ddPCR copy number is low. R’s lm module was used to calculate the line of linear regression for plotting purposes. Given that ddPCR measures gene presence and FISH measures gene absence, for purposes of plotting FISH-ddPCR comparison, the negative of FISH count was used (ie. FISH count multiplied by -1). Fisher’s exact test was used to determine significance of concordance between IHC and FISH results, and Cohen’s kappa was used to measure this concordance. Fisher’s exact test was used to determine the association between FISH and ddPCR results. Sensitivity was calculated as number of true positives divided by the sum of true positives and false negatives. Specificity was calculated as number of true negatives divided by the sum of true negatives and false positives.

## Results

### Samples Include All MPM Types: Epithelioid, Sarcomatoid, and Biphasic

We initially included 85 patients in the study and for 75 of these there was sufficient quantity of FFPE sample for biomarker analysis. MPM subtype was classified according to the current WHO classification of mesothelioma (6). There were 37 (49%) epithelioid, 26 (45%) biphasic and 12 (16%) sarcomatoid mesothelioma patient samples included. Patient median age at operation (during which tissue sample was taken) was 65 with a range of 51 to 93. All patients underwent pleurectomy/decortication (P/D) from which tissue samples were taken for analysis. Patients received no neoadjuvant chemotherapy treatment prior to the P/D procedure. Patient demographics are detailed in **Table 1** below. MTAP and CDKN2A were successfully analyzed in more than 60% of the cases. Some samples that were collected over 10 years prior to our experiment were found to be compromised on the basis that they did not provide sufficient reference copy numbers to differentiate the deletion of CDKN2A or MTAP and were therefore excluded from the experiment. Samples that met adequate quality control produced sufficient detectable copies for the reference gene and deleted MTAP or CDKN2A (tests performed on each sample are listed in **Supplementary Table 2**).

**Table 1:** Mesothelioma patient demographics.

Gender	Male	58 (77%)
	Female	17 (23%)
Median age (range)	Median	65 (51–93)
MPM type		
	Epithelioid	37 (49%)
	Biphasic	26 (45%)
	Sarcomatoid	12 (16%)

### IHC Shows MTAP Protein Loss in 48% of MPM Patient Samples

**Figure 1:**
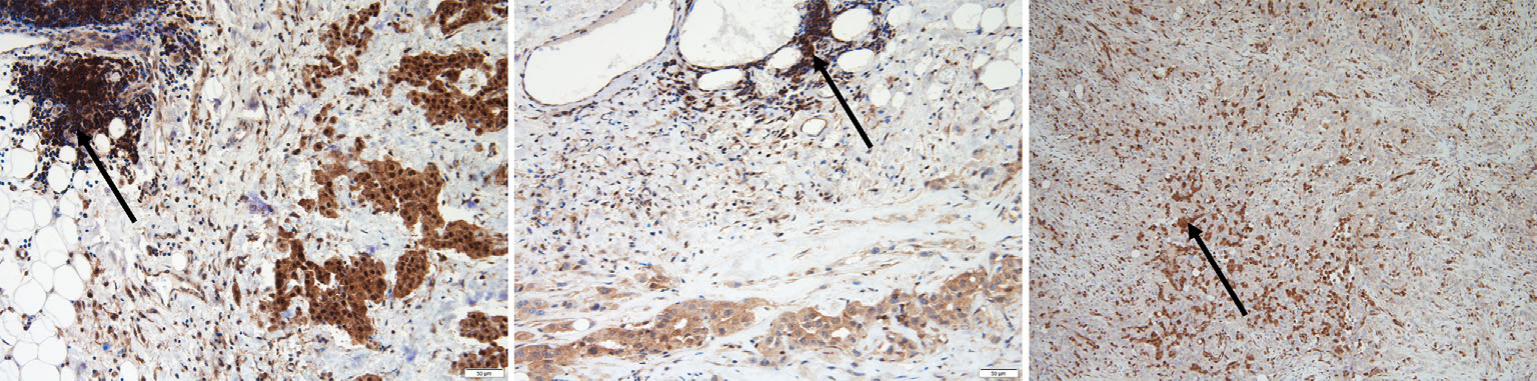
A representative image showing epithelioid malignant pleural mesothelioma (MPM) with methylthioadenosine phosphorylase (MTAP) immunohistochemistry staining. Retained MTAP cytoplasmic expression (left); partial loss of MTAP cytoplasmic staining (middle) and complete loss of MTAP cytoplasmic staining (right); all with internal control lymphocytes with retained cytoplasmic staining for comparison (with arrows).

We first analyzed FFPE samples *via* IHC analysis to study MTAP protein expression. We also analyzed 10 RMH samples for MTAP IHC expression and all were MTAP IHC retained as expected (**Supplementary Table 4B**). Among the 75 samples, one did not achieve good FFPE tissue quality for analysis, one did not reveal any residual tumor after IHC was performed and one sample was poorly adherent to the slides, and thus results could not be obtained. Of the 72 remaining patient samples analyzed for MTAP protein expression, MTAP loss was found in 35 samples (49%), 36 samples (50%) showed retained MTAP, and 1 (1%) showed partial loss of MTAP expression (**Supplementary Table 3**). Representative images of MTAP cytoplasmic loss, partial loss and retained MTAP expression are shown in **Figure 1**. Details for MTAP expression and tumor content of each sample are listed in **Supplementary Table 3**. It is important to note that in some cases, there are some samples with <10% of tumor that shows MTAP IHC loss, but the numbers of these cases are small (3/72) in the entire analyzed cohort. This could reflect tumor heterogeneity staining and may not be truly representative of the staining pattern of the entire tumor.

### FISH Shows Co-Deletion of MTAP and CDKN2A in 95% of Cases

**Figure 2:**
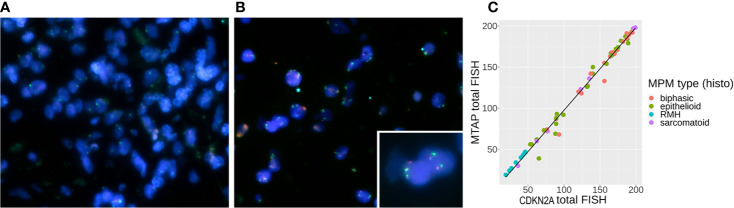
**(A)** Tri-color [methylthioadenosine phosphorylase (MTAP) – aqua, CDKN2A – red, CEPT9 – green] probe fluorescence *in situ* hybridization (FISH) staining showing genomic loss of MTAP and CDKN2A in mesothelioma cells from an operated malignant pleural mesothelioma (MPM) (P/D) sample, **(B)** FISH staining showing positive signals from all three probes in the reactive mesothelial hyperplasia (RMH) sample, where the inset shows two pairs of retained red CKDN2A and aqua MTAP genes. **(C)** Correlation between CDKN2A FISH results and MTAP FISH results with line of linear regression in black (slope = 1.007, y-intercept = −3.049, R^2^ = 0.989).

We initially included 72 FFPE MPM samples. However, some were not of suitable quality for FISH, as the tissue sections did not survive the hybridisation step. We were able to confidently obtain FISH results for 56 FFPE MPM samples comprising 28 (50%) epithelioid, 21 (37.5%) biphasic, and 7 (12.5%) sarcomatoid cases. A representative image of CDKN2A and MTAP tricolor FISH staining is shown in **Figures 2A, B**. For each sample, counts for homozygous cells, hemizygous cells or no loss of MTAP or CDKN2A were tallied (details listed in **Supplementary Table 4A**). The 10 RMH samples were also analyzed for MTAP and CDKN2A. All ten samples showed no homozygous loss of both genes (**Supplement Table 4B**), however 4 cases showed hemizygous loss. These hemizygous losses could represent truncation artifact but also could represent true CDKN2A hemizygous heterogeneous loss in reactive mesothelial hyperplasia samples. This would explain previous reports in the literature in only using homozygous CDKN2A loss as significant in differentiating MPM from RMH (9, 20, 21). We took the opportunity to calculate the sensitivity and specificity of CDKN2A homozygous loss by FISH in differentiating MPM from RMH (**Supplementary Table 5**). We used our previously determined cut-off threshold, that is, when more than 15% of cells show homozygous deletion by FISH result, then the MPM is considered to have homozygous deletion of the genomic region analyzed. For the 56 MPM samples, 79% (44/56) had homozygous deletion, 18% (10/56) had hemizygous deletion, and 3% (2/56) had no deletion of CDKN2A (**Table 2**). We also correlated the results of CDKN2A homozygous loss with MPM subtypes (**Table 3**). With respect to MTAP gene status of these 56 cases, 77% (43/56) had homozygous deletion, 18% (10/56) had hemizygous deletion, and 5% (3/56) had no deletion of MTAP (**Table 4A**). All 10 RMH samples showed no homozygous loss of CDKN2A and MTAP by FISH analysis (**Supplementary Table 4B**). The calculated sensitivity and specificity of homozygous CDKN2A loss by FISH is 78% and 100% respectively (**Supplementary Table 5**). For 53 of the 56 cases (95%), there was deletion (either homozygous or hemizygous) of either or both MTAP and CDKN2A (**Table 4B**). For 50 of the 53 (94%) cases that displayed deletion, the results for MTAP and CDKN2A were the same in each case, that is, showing both homozygous or hemizygous co-deletion, (Fisher test p-value = 2.2e-16 showing that the FISH results for MTAP and CDKN2A are co-dependent). For 42 of the 44 cases (95%), MTAP and CDKN2A were homozygous co-deleted. For 2 of the 44 CDKN2A homozygous deleted cases (5%), the two genes were not deleted in tandem (co-deleted) with MTAP (**Supplementary Table 4A**). This form of discrepancy has been described previously (12).

**Table 2:** Homozygous, hemizygous and no loss of CDKN2A detected by fluorescence *in situ* hybridization (FISH) in malignant pleural mesothelioma (MPM) cases.

CDKN2A Homozygous Loss	CDKN2A Hemizygous Loss	CDKN2A No Loss	Total
79% (44/56)	18% (10/56)	3% (2/56)	100% (56/56)

**Table 3:** Proportion of CDKN2A homozygous loss according to malignant pleural mesothelioma (MPM) subtype.

MPM Subtype	CDKN2A Homozygous Loss
Epithelioid	68% (19/28)
Sarcomatoid	71% (5/7)
Biphasic	95% (20/21)
Total cases homozygous loss analysed by FISH	78% (44/56)

**Table 4A:** Homozygous, hemizygous and no loss of methylthioadenosine phosphorylase (MTAP) detected by fluorescence *in situ* hybridization (FISH) in malignant pleural mesothelioma (MPM) cases.

MTAP Homozygous Loss	MTAP Hemizygous Loss	MTAP No Loss	Total
77% (43/56)	18% (10/56)	5% (3/56)	100% (56/56)

**Table 4B:** Comparison of fluorescence *in situ* hybridization (FISH) results for detecting methylthioadenosine phosphorylase (MTAP) and CDKN2A deletion in malignant pleural mesothelioma (MPM) cases.

	CDKN2A Hemizygous loss	CDKN2A Homozygous loss*	CDKN2A no loss	Total
MTAP Hemizygous loss	8	2	0	10
MTAP Homozygous loss*	1	42	0	43
MTAP no loss	1	0	2	3
Total	10	44	2	56

*Not shown: 10 RMH samples showed no homozygous loss of CDKN2A or MTAP by FISH.

As shown in **Figure 2C**, the count of CDKN2A loss is highly correlated with the count of MTAP loss (Spearman’s rank correlation rho = 0.99 with p-value = 2.2e-16 between the two genes’ total FISH counts, and Spearman’s rank correlation rho = 0.99 and 0.98 respectively for homozygous counts and hemizygous counts with p-value = 2.2e-16 in both cases). **Table 4** shows counts and comparison of MTAP and CDKN2A loss by FISH analysis (details in **Supplementary Tables 4A, B**).

### ddPCR Analysis Shows Co-Deletion of MTAP and CDKN2A in 82% of Cases

We performed ddPCR analysis using FFPE samples. Seventy five cases were initially included for analysis but of these cases, 43 were acceptable for MTAP analysis, and only 39 were suitable for CDKN2A analysis due to satisfactory quality control (QC) criteria (E2 count greater than 100) (details provided in **Supplementary Table 6**). Again, we took the opportunity to calculate the sensitivity and specificity of CDKN2A homozygous loss by ddPCR in differentiating MPM from RMH (**Supplementary Table 7**).

**Figure 3:**
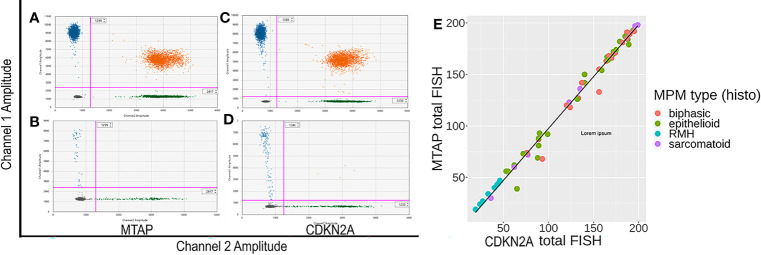
**(A–D)** Representative images of methylthioadenosine phosphorylase (MTAP) and CDKN2A droplet digital PCR (ddPCR) analyses. **(A, C)** are control samples with both targeted region (MTAP or CDKN2A) retained. **(B, D)** are patient formalin fixed paraffin embedded (FFPE) samples showing all positive populations are detectable. In samples having genomic (MTAP or CDKN2A) deletion, the top left population (blue) disappears while the reference population (green) remains. **(E)** Correlation between CDKN2A ddPCR results and MTAP ddPCR results for MPM and negative control normal pleural tissue samples, with line of linear regression in black (slope =0.8193, y-intercept = 0.2030, R^2^ = 0.875).

**Figures 3A–D** show representative images of ddPCR population detection. Analysis results for MTAP demonstrated that 58% (25/43) were homozygous deletion and 42% (18/43) were hemizygous deletion, with no cases showing no loss (**Table 5**). Analysis results for CDKN2A showed that 72% (28/39) were homozygous deletion, 26% (10/39) were hemizygous deletion, and 2% (1/39) had retained CDKN2A (**Table 6**). All 12 normal pleural tissue samples showed retention of CDKN2A and MTAP by ddPCR analysis. In MPM, the sensitivity and specificity of CDKN2A homozygous loss is 72% and 100% respectively, as determined by ddPCR analyses (**Supplementary Table 7**). The 39 cases analyzed for CDKN2A also yielded concurrent MTAP analyzed results. There was deletion (homozygous or hemizygous) of either or both CDKN2A and MTAP in 97% (38/39) of the cases. For 87% (33/38) of the cases that demonstrated deletion, the results for CDKN2A and MTAP were the same in each case, that is, both homozygous co-deletion, or both hemizygous co-deletion. 82% (23/28) of cases displayed homozygous co-deletion of MTAP and CDKN2A (**Table 7**) (Fisher test p-value = 1.204e-15 showing that the ddPCR results for MTAP and CDKN2A are dependent on each other). Our ddPCR copy number results for MTAP and CDKN2A on MPM and non-MPM lung tissue are highly correlated (Spearman’s rank correlation rho = 0.90, p-value = 2.2e-16), as shown in **Figure 3E**. For 1 of the 39 cases (2%), a hemizygous MTAP deletion without an associated loss of CDKN2A was detected. Thus, similar to our FISH results, our ddPCR results also showed frequent MTAP and CDKN2A co-deletion. **Supplementary Table 6** contains detail information of ddPCR of all samples.

**Table 5:** Homozygous, hemizygous and no loss of methylthioadenosine phosphorylase (MTAP) detected by droplet digital PCR (ddPCR) in malignant pleural mesothelioma (MPM) samples.

MTAP Homozygous Loss	MTAP Hemizygous Loss	MTAP No Loss	Total
58% (25/43)	42% (18/43)	0%	100% (43/43)

**Table 6:** Homozygous, hemizygous and no loss of CDKN2A detected by droplet digital PCR (ddPCR) in malignant pleural mesothelioma (MPM) samples.

CDKN2A Homozygous Loss	CDKN2A Hemizygous Loss	CDKN2A No Loss	Total
72% (28/39)	26% (10/39)	2% (1/39)	100% (39/39)

**Table 7:** Comparison of droplet digital PCR (ddPCR) results for detecting methylthioadenosine phosphorylase (MTAP) and CDKN2A deletion in malignant pleural mesothelioma (MPM) cases.

	CDKN2A Hemizygous loss	CDKN2A Homozygous loss	CDKN2Ano loss	Total
MTAP Hemizygous loss	10	5	1	16
MTAP Homozygous loss	0	23	0	23
MTAP no loss	0	0	0	0
Total	10	28	1	39

### Concordance Between ddPCR and FISH for MTAP and CDKN2A Biomarkers

**Figure 4:**
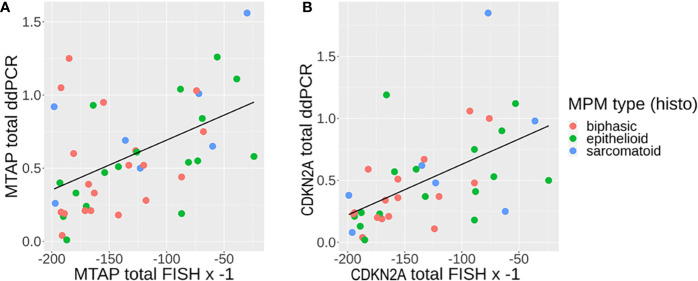
**(A, B)** Correlation between fluorescence *in situ* hybridization (FISH) (multiplied by −1, since any one cell containing genomic loss will count as 1) and droplet digital PCR (ddPCR) results (genomic loss will show no detection) for **(A)** MTAP and **(B)** CDKN2A, with line of linear regression in black [A: methylthioadenosine phosphorylase (MTAP): slope = 0.003411, y-intercept = 1.034011, R^2^ = 0.2404; B: CDKN2A: slope = 0.004099, y-intercept = 1.040047, R^2^ = 0.302].

We then proceeded to determine the concordance between ddPCR and FISH in detecting the genetic status of CDKN2A and MTAP. 52% (39/75) of the overall case cohort analyzed had concurrent CDKN2A and MTAP results by ddPCR and FISH available for concordance study (details in **Supplementary Table 8**). Both FISH and ddPCR provide counts that can be compared on a continuous scale. The correlation between FISH and ddPCR for MTAP is good (Spearman’s rank correlation rho = -0.48, p-value = 0.001). It is negative, as expected for a correlation between FISH and ddPCR given that gene deletion will result in a high number count for FISH and low number count for ddPCR, and vice versa for gene retention. The correlation between FISH and ddPCR for CDKN2A is even higher (Spearman’s rank correlation rho = −0.59, p-value = 8.39e-05). The correlations are plotted in **Figures 4A, B**. Our results show that the concordance of ddPCR with the gold standard FISH is 93% and 92% for detecting MTAP and CDKN2A loss (where loss is defined as either homozygous or hemizygous loss) respectively (**Tables 8A**, **B**). The concordance between ddPCR and FISH for homozygous CDKN2A deletion is 92% (23/25). Almost all of our mesothelioma cases exhibited a loss of MTAP and CDKN2A. None of the 43 cases analyzed for MTAP by ddPCR showed no loss (**Table 5**). One case analyzed by ddPCR showed no loss of CDKN2A, but was homozygous deleted by FISH (**Table 8B**). It is useful to determine the presence of losses of MTAP and CDKN2A, however in terms of the diagnosis of MPM, it is more pertinent to specifically identify homozygous loss (12).

**Table 8A:** Comparison of droplet digital PCR (ddPCR) and fluorescence *in situ* hybridization (FISH) results for detecting methylthioadenosine phosphorylase (MTAP) homozygous and hemizygous loss. (details in **Supplementary Table 8**).

MTAP ddPCR scoring	FISH homozygous MTAP loss	FISH hemizygous MTAP loss	FISH MTAP no loss	Concordance between ddPCR and FISH	Discordance between ddPCR and FISH
Homozygous Loss (n=25)	23	2	0	92% (23/25)	8% (2/25)
Hemizygous Loss (n=18)	9	6	3	33% (6/18)	67% (12/18)
Homozygous & hemizygous loss (n=43)	32	8	3	93% (40/43)	7% (3/43)
No loss (n=0)*	Not assessable	Not assessable	Not assessable	Not assessable	Not assessable

*There are no cases of retained (no loss) MTAP analyzed by ddPCR hence concordance with FISH is not assessable.

**Table 8B:** Comparison of droplet digital PCR (ddPCR) and fluorescence *in situ* hybridization (FISH) results for detecting CDKN2A homozygous and hemizygous loss.

cdkn2a ddPCR scoring	FISH CDKN2A homozygous loss	FISH CDKN2A hemizygous loss	FISH CDKN2A no loss	Concordance between ddPCR and FISH	Discordance between ddPCR and FISH
Homozygous Loss (n=28)	26	2	0	92% (26/28)	8% (2/28)
Hemizygous Loss (n=10)	3	5	2	50% (5/10)	50% (5/10)
Homozygous & hemizygous loss (n=38)	29	7	2	95% (36/38)	5% (2/38)
No Loss (n=1)	1	0	0	Nil	100% (1/1)

### Concordance of MTAP IHC Results With FISH and ddPCR for MTAP Gene Analysis, and MTAP IHC With ddPCR and FISH CDKN2A Homozygous Deletion

We measured how well MTAP IHC results were concordant with FISH and ddPCR results for the MTAP and CDKN2A gene. FISH and ddPCR measure genomic DNA features whereas IHC measures the resulting protein. In **Table 9A** we present the counts of concordance for MTAP IHC and MTAP FISH, and **Table 9B** for MTAP IHC and CDKN2A FISH. In **Table 9C** and **D** we present the counts of concordance for MTAP IHC and CDKN2A and MTAP ddPCR respectively. The concordance of MTAP IHC with MTAP FISH as a test was 71% (**Table 9A**). For the concordance of MTAP IHC with CDKN2A FISH overall as a test was 70% (**Table 9B**). The concordance of MTAP IHC with MTAP and CDKN2A using ddPCR as a test overall was 67% and 69% respectively (**Tables 9C** and **9D**). In terms of concordance comparison of MTAP IHC loss with MTAP and CDKN2A FISH homozygous deletion, MTAP IHC is inferior to ddPCR; 65% vs 92% for MTAP (**Tables 9A** and **8A**) and 64% vs 92% for CDKN2A (**Tables 9B** and **8B**). The ability of IHC to distinguish between homozygous loss and retention of MTAP as reported by FISH is weak (Cohen’s kappa = 0.3225) and is not statistically significant (Fisher test p-value = 0.00027). IHC is not able to distinguish between loss and retention of MTAP as reported by ddPCR. Interestingly, MTAP IHC is retained for 14% (8/56) of cases harboring both hemizygous co-deleted CDKN2A and MTAP (**Supplementary Table 8**). The three cases had non-tandem deletion of MTAP and CDKN2A also showed MTAP IHC retention (**Supplementary Table 8**). In addition, as previously described, MTAP IHC is a good surrogate method in detecting the homozygous loss of CDKN2A in MPM (13, 22). We re-validated this finding in our study. Using FISH as the gold standard method for CDKN2A genetic analysis, the sensitivity and specificity of MTAP IHC is 64% and 100% respectively (**Supplementary Table 9**). Using ddPCR as the test for the genetic analysis, the sensitivity is 68% and 100% respectively (**Supplementary Table 10**).

**Table 9A:** Comparison of methylthioadenosine phosphorylase (MTAP) immunohistochemistry (IHC) and MTAP fluorescence *in situ* hybridization (FISH) homozygous and hemizygous deletion, and no loss (retention).

MTAP FISH scoring	MTAP IHC loss	MTAP IHC retained	Concordance between MTAP IHC and MTAP FISH hemizygous loss, homozygous loss and no loss
Homozygous Loss (n=43)	28 (includes 1 case of partial loss)	15	65% (28/43)
Hemizygous Loss (n=10)	1	9	90% (9/10)
No Loss (n=3)	0	3	100% (3/3)
Overall Concordance of MTAP IHC and MTAP FISH as a test			71% (40/56)

**Table 9B:** Comparison of methylthioadenosine phosphorylase (MTAP) immunohistochemistry (IHC) and CDKN2A fluorescence *in situ* hybridization (FISH) homozygous and hemizygous deletion, and no loss (retention).

CDKN2A FISH scoring	MTAP IHC loss	MTAP IHC retained	Concordance between MTAP IHC and CDKN2A FISH hemizygous loss, homozygous loss and no loss
Homozygous Loss (n=44)	28 (includes one case of partial loss)	16	64% (28/44)
Hemizygous Loss (n=10)	1	9	90% (9/10)
No Loss (n=2)	0	2	100% (2/2)
Overall Concordance MTAP IHC and CDKN2A FISH as a test			70% (39/56)

**Table 9C:** Comparison of methylthioadenosine phosphorylase (MTAP) immunohistochemistry (IHC) and MTAP droplet digital PCR (ddPCR) homozygous and hemizygous deletion, and no loss (retention).

MTAP ddPCR results	MTAP IHC loss	MTAP IHC retained	Concordance between MTAP IHC and MTAPddPCR Homozygous loss
Homozygous Loss (n=25)	17	8	68% (17/25)
Hemizygous Loss (n=18)	6	12	66% (12/18)
No Loss (n=0)	0	0	
Overall Concordance MTAP IHC and MTAP ddPCR as a test			67% (29/43)

## Discussion

MPM is an aggressive malignancy and typically a diagnostic challenge. Currently, clinical diagnosis requires skilful and time-consuming pathology input and the use of extensive immunohistochemical biomarker testing for definitive diagnosis. Invasive procedures are currently used to obtain the gold standard tissue biopsy to help in the definitive diagnosis of MPM. In addition to the cost, risk, and time delays associated with surgical procedures, the pathological diagnosis also requires experienced and labor-intensive pathology testing. Thus, currently there is much interest and activity in biomarker development and emerging technology for biomarker detection of MPM. We have previously shown that ddPCR is a useful platform whose results are comparable to labor-intensive FISH analysis in MPM (7). The present study is focussed on two biomarkers, CDKN2A and MTAP, that are adjacent to each other on chromosome 9p21. Previous studies have shown that in a majority of MPM cases, CDKN2A and MTAP are co-deleted (12) and that loss of MTAP IHC is an additional useful routine biomarker in diagnosing MPM (8, 13, 14).

In this study of 75 MPM cases, we carried out IHC, FISH and ddPCR analysis for MTAP; and FISH and ddPCR analysis for CDKN2A. Our results show that co-deletion of MTAP and CDKN2A is a frequent event as measured by FISH and ddPCR (Spearman’s rank correlation rho = 0.99 and 0.93, and p-value = 2.2e-16 for both FISH and ddPCR). Our study thus reconfirms the previous report by Ileal et al., that both MTAP and CDKN2A are frequently co-deleted in MPM (12). However, our cases of co-deletion by FISH is slightly higher at 95% compared to Ileal et al. (91%), but lower by ddPCR (82%).

Our study is the first study in the English literature that investigates the concordance between ddPCR and FISH in detecting the loss of CDKN2A and MTAP in MPM, and therefore a potentially useful alternative method in the diagnostic algorithm of MPM. Both FISH and ddPCR are sensitive methods that provide counts on the continuous scale. Our results showed that ddPCR and FISH are highly correlated for analysis of CDKN2A and MTAP genomic loss (Spearman’s rank correlation rho = -0.59 and -0.48, p-value = 8.39e-05 and 0.001 for CDKN2A and MTAP respectively). By ddPCR alone, the sensitivity is 72% and 100% specific for CDKN2A homozygous loss in MPM. This is similar to the sensitivity and specificity of 79% and 100%, respectively, for homozygous loss in MPM detected by FISH in our study. Our sensitivity and specificity rates by ddPCR is comparable to previous reports in the literature using FISH (20, 21, 23) where the sensitivities ranged from 59% to 88%, but the specificity was 100%, thus suggesting that ddPCR is a comparable alternative method in the diagnosis of MPM.

Our study shows a concordance rate of 92% between ddPCR and FISH in detecting CDKN2A homozygous loss, and 92% concordance for overall losses (including both hemizygous and homozygous loss). There were 14 cases that were discordant between ddPCR and FISH for analyzing MTAP (**Table 8A**), and 8 discordant cases when analyzing CDKN2A (**Table 8B**). Seven ddPCR cases shared discordance with FISH on analysis of both MTAP and CDKN2A. After careful analysis of discordant cases, discordant cases could be grouped into three broad categories. They are marginal ddPCR counts, marginal FISH counts and tumor heterogeneity. Marginal count cases either for ddPCR or FISH were cases that marginally passed the cut off threshold and be classified into that discordant group, which otherwise would have been concordant. Taking case 190093 as an example, ddPCR was hemizygous but FISH was homozygous. However, the ddPCR count is just above the cut off for hemizygous (0.44 for MTAP and 0.48 for CDKN2A, homozygous range 0 to 0.4; hemizygous range 0.4 to 1.5, please see ddPCR material and methods), while the FISH count was well above the cut off (>15%). Therefore, this case was marginal ddPCR count and would have otherwise been in the homozygous category and would have been concordant with FISH. Regarding tumor heterogeneity, we have shown in our study that MTAP and CDKN2A genomic pattern when analyzed by FISH can be a mixture of retained (no loss), homozygous or hemizygous loss signal patterns within one tumor (**Supplementary Table 8**). Therefore, the final classification of the genomic loss pattern of that tumor is based upon the specific signal pattern by FISH that meets the acceptable threshold to be classified as no loss, hemizygous or homozygous loss. Although there is an attempt to enumerate at least 100 tumor cells collectively in various areas of the tumor during FISH analysis, this could still be subjected to selection bias in the analyzed area since it is impractical to enumerate all the tumor cells in the entire surgical biopsy specimen by FISH, and the area analyzed would still be less compared to the entire tissue specimen when analyzed by ddPCR. However, ddPCR analyses the entire FFPE section for DNA isolation, and threshold cut offs are based upon the amount of tumor cell percentage present in the entire tissue section, therefore potentially eliminating selection bias of the tumor area assessed. Out of the seven cases that showed discordance of both MTAP and CDKN2A between ddPCR and FISH, four cases (190049, 190080, 190088, 190092) were attributed to be because of tumor heterogeneity, two cases (190089 and 190093) were marginal ddPCR threshold and one case (190042) was marginal FISH threshold. Out of the other seven discordant MTAP only cases, three cases (190075, 190081, and 190099) were attributed to tumor heterogeneity, three cases (190106, 190118, 190120) were due to marginal ddPCR count threshold and one case (190043) was due to marginal FISH count threshold. The one other discordant CDKN2A only case (190072) was due to marginal ddPCR count threshold. The presence of these discordant cases raises a potential point of weakness in our study. In ddPCR analysis, the entire tissue section encompassing both tumor and non-tumor cell population are analyzed. This could potentially dilute the tumor sample DNA. In order to increase the purity of the sample analyzed, potentially laser capture microdissection (LCM) may be employed to isolate as much tumor cell population as possible. However, this study aims to be translated to real world experience, and performing LCM in every case is not time or cost effective.

Our study also analyzed MTAP gene loss status by FISH and ddPCR. In addition to the publications by Ileal et al. and Krasinskas et al. (12, 24), this current report is one of the few in the literature to analyze and re-validate MTAP genomic loss status in MPM. Many recent studies have used MTAP IHC to increase the sensitivity in diagnosing mesothelioma (8, 13, 14, 22, 25), since previous studies indicated that p16 IHC has poor concordance with homozygous CDKN2A loss in MPM (9, 14). The sensitivities of MTAP IHC in these reports ranged from 33% to 78%, with specificity being 100% in all these studies. Our results also show similar MTAP IHC sensitivity and specificity of 48% and 100%, respectively in MPM cases. But when focusing specifically on MPM cases with only CDKN2A homozygous loss, MTAP IHC sensitivity in our study is 64% and 68% when the genetic losses were detected by FISH and ddPCR, respectively. This is also similar to these recent publications (8, 13, 14, 22, 25) with sensitivities ranging from 68% to 82% when assessing MTAP IHC with CDKN2A homozygous loss MPM cases. The concordance of MTAP IHC with homozygous loss, hemizygous loss and retention of CDKN2A as an overall test with FISH is 70% (**Table 9B**), and 69% with ddPCR (**Table 9D**) in our study. This is similar compared to Berg et al. (71%) (25), but inferior to Berg et al. (84%) (8). Although, it is worth mentioning that our current study has a much larger case cohort. We included cases of MTAP IHC retained with CDKN2A and MTAP hemizygous deletion as a concordant result because of the existence of another allele, which could lead to MTAP IHC positive staining. Since we assessed MTAP genomic loss status, we have an advantage over recent reports (8, 13) in suggesting an explanation of cases with MTAP IHC positivity, but MTAP homozygous loss. This could be due to tumor heterogeneity and/or antibody which is similar to previous studies on HER2 IHC and FISH discordance (26, 27). As mentioned, 95% of our cases had homozygous co-deletion of MTAP and CDKN2A. Chapel et al. (13) have suggested that “false negative results (MTAP IHC positive staining)” when using MTAP IHC were due to MTAP IHC positivity in tumor-infiltrating lymphocytes and histiocytes leading to interpretation of positive MTAP IHC in MPM cells, when there was CDKN2A homozygous loss by FISH. In our study we showed that MTAP IHC positivity was in 16% (9/56) MTAP hemizygous loss cases and 27% (15/56) in MTAP homozygous loss cases (**Supplementary Table 8**). In regards to MTAP IHC positivity in cases with MTAP hemizygous loss, we suggest that this finding would seem logical as retention of the other MTAP gene could potentially lead to protein transcription and translation, and hence IHC being detected. The explanation of MTAP IHC positivity in MTAP homozygous loss cases could be attributed to the design of FISH probes, ddPCR primers and MTAP antibody. The MTAP gene is about 64Kb in length whereas the MTAP FISH probe (~400bp) and ddPCR primers (96bp) are much shorter than the entire MTAP genome. Potentially, only part of the MTAP gene is deleted which are detected by these particular shorter FISH probe or ddPCR primers, but other protein coding portions of the gene is retained. This finding is consistent with general understanding of mechanisms mediating or relating to incomplete genomic translation, and have been described in other malignancies (28–30). As such a partially expressed MTAP protein may be detected by the antibody leading to a “false negative” MTAP IHC result. Of note, our one case where there was MTAP IHC partial cytoplasmic loss (diminished IHC expression) also showed homozygous loss of CDKN2A by FISH.

**Table 9D:** Comparison of methylthioadenosine phosphorylase (MTAP) immunohistochemistry (IHC) and CDKN2A droplet digital PCR (ddPCR) homozygous and hemizygous deletion, and no loss (retention).

CDKN2A ddPCR results	MTAP IHC loss	MTAP IHC retained	Concordance between MTAP IHC and CDKN2AddPCR Homozygous loss
Homozygous Loss (n=28)	19	9	68% (19/28)
Hemizygous Loss (n=10)	3	7	70% (7/10)
No Loss (n=1)	0	1	100% (1/1)
Overall Concordance MTAP IHC and CDKN2A ddPCR as a test			69% (27/39)

In conclusion, the results of our study provide evidence that ddPCR is a robust method that shows good concordance with the current gold standard FISH in detecting MTAP and CDKN2A homozygous deletion in MPM. ddPCR also shows similar sensitivity and specificity as FISH in differentiating MPM from RMH. This is a welcoming result because FISH is labor-intensive compared to ddPCR, and although ddPCR requires trained personnel for DNA isolation, FISH analysis requires a more advanced level of technique and experience to score samples. Furthermore, FISH analysis is also time-consuming, with the analysis being limited to one case at a time. This is in contrast to ddPCR analysis where it is less biased, does not require histomorphologic assessment, and more time efficient as it is able to analyze multiple samples in one setting. However, the advantage of FISH over ddPCR is the ability to analyze tumor cell genomic heterogeneity. This is because each tumor cell is analyzed individually for the signal patterns such as losses, gains, polysomies or amplification. Our results also suggest that ddPCR could potentially be used as an alternative diagnostic method on cytology cell block samples where scattered individual tumor cells and cell clusters are among a background of inflammatory cells. ddPCR could also potentially be used on blood-derived tumor circulating DNA, instead of FISH on tissue sections, which are often obtained through more invasive procedures. Undoubtedly, further studies will be required to explore this possibility. We anticipate that the results of this study will yield essential data that may lead to the clinical utility of ddPCR as an alternative to FISH in detecting the loss of MTAP and CKDN2A in MPM tissue samples, and potentially facilitate the development of ddPCR as a testing platform for the analysis of diagnostic specimens obtained through less invasive methods.

## Data Availability Statement

The original contributions presented in the study are included in the article/**Supplementary Material**. Further inquiries can be directed to the corresponding author.

## Ethics Statement

The studies involving human participants were reviewed and approved by Human Research Ethics Committees at Concord Repatriation General Hospital (HREC/11/CRGH/75 approved since 2011). The patients/participants provided their written informed consent to participate in this study.

## Author Contributions 

KL and YC conceived the project. YC carried out the experiments in this study including ddPCR assay design, ddPCR analysis, preparation of cell blocks, sectioning, DNA isolation, drafting and editing the manuscript. KL is an experienced pathologist who provided professional scoring of MPM biomarkers using IHC staining and FISH analysis and editing of the manuscript. ER provided conceptual input and edited the manuscript as well as statistical analysis of all data, CC carried out sectioning and staining for IHC and FISH, MY, LZ, VA, BJ, and T-kY organized FFPE samples for DNA isolation. BM is a cardiovascular surgeon who operated on and collected all patient samples for this study. KT contributed to manuscript preparation. AL and SK contributed to collection of all the clinical samples and clinical backgrounds. All authors contributed to the article and approved the submitted version.

## Funding

This project is partially funded by Australian Academy of Science – Regional Collaborations Programme grand (2019).

## Conflict of Interest

The authors declare that the research was conducted in the absence of any commercial or financial relationships that could be construed as a potential conflict of interest.
